# *Plasmodium vivax*: who cares?

**DOI:** 10.1186/1475-2875-7-S1-S9

**Published:** 2008-12-11

**Authors:** Mary R Galinski, John W Barnwell

**Affiliations:** 1 Emory Vaccine Center and Yerkes National Primate Research Center, Division of Infectious Diseases, Department of Medicine, School of Medicine, Emory University, Atlanta, GA, USA; 2 Malaria Branch, Division of Parasitic Diseases, National Center for Zoonotic, Vector-Borne and Enteric Diseases, the Centers for Disease Control and Prevention, Atlanta, GA, USA

## Abstract

More attention is being focused on malaria today than any time since the world's last efforts to achieve eradication over 40 years ago. The global community is now discussing strategies aimed at dramatically reducing malarial disease burden and the eventual eradication of all types of malaria, everywhere. As a consequence, *Plasmodium vivax*, which has long been neglected and mistakenly considered inconsequential, is now entering into the strategic debates taking place on malaria epidemiology and control, drug resistance, pathogenesis and vaccines. Thus, contrary to the past, the malaria research community is becoming more aware and concerned about the widespread spectrum of illness and death caused by up to a couple of hundred million cases of vivax malaria each year. This review brings these issues to light and provides an overview of *P. vivax *vaccine development, then and now. Progress had been slow, given inherent research challenges and minimal support in the past, but prospects are looking better for making headway in the next few years. *P. vivax*, known to invade the youngest red blood cells, the reticulocytes, presents a strong challenge towards developing a reliable long-term culture system to facilitate needed research. The *P. vivax *genome was published recently, and vivax researchers now need to coordinate efforts to discover new vaccine candidates, establish new vaccine approaches, capitalize on non-human primate models for testing, and investigate the unique biological features of *P. vivax*, including the elusive *P. vivax *hypnozoites. Comparative studies on both *P. falciparum *and *P. vivax *in many areas of research will be essential to eradicate malaria. And to this end, the education and training of future generations of dedicated "malariologists" to advance our knowledge, understanding and the development of new interventions against each of the malaria species infecting humans also will be essential.

## Background

Malaria vaccine research and development efforts, since the early 1980s, have almost exclusively been focused on *Plasmodium falciparum*, the most prevalent and most deadly of the human malaria parasite species [1]. When first approached to write this review with a focus on *Plasmodium vivax *vaccines, a somewhat whimsical thought was: Who cares? A relatively small group of scientists involved in research on *P. vivax *for over 20 years have been repeatedly faced with the challenge of making arguments to funding bodies to support research on what has been viewed, most certainly inappropriately, as a "benign" parasite. The world is now recognizing that the 'benign' designation has been an unfortunate misnomer used widely in the literature [2,3] and it is satisfying to finally witness a credible shift in concern and a surge of attention on *P. vivax*.

So, who does care and what has caused this shift? First of all, certainly the people who live in places across the globe with the day-to-day threat of being infected with this parasite care passionately. Patient's anecdotes repeatedly indicate that being sick with *vivax *malaria is terrible and makes one '*feel like they are going to die*'. *Vivax *malaria even today ranges from temperate through the subtropical and tropical zones of the world exhibiting an array of adaptations that enable this parasite to exist in widely varying ecological and climatic conditions [4,5]. Since estimates for the number of *vivax *malaria cases have ranged from a minimum of 35,000,000 to 80,000,000, or perhaps a couple of hundred million more, the morbidity from this disease cannot be considered inconsequential, despite its moniker of 'benign tertian malaria' [3,6-8]. Given a propensity towards significant and severe anaemia, thrombocytopaenia, violent paroxysms and fevers of 40°C to 41.6°C that, if untreated, can last for weeks, uncomplicated *vivax *malaria is a disease with serious morbidity [7]. Furthermore, the recent publication of several well-documented studies have highlighted and validated older anecdotal evidence that *P. vivax *infections, much like *P. falciparum*, can frequently cause severe and complicated clinical disease syndromes, that may result in death [9-13]. These elements alone argue for greater consideration of the inclusion of *vivax *malaria in research portfolios, provision of targeted research support that will facilitate the study of this human malaria, and in planning of *vivax *malaria control efforts by policy makers and funding bodies.

A second simple truth regarding the cause for the shift in who cares about *vivax *malaria came about when in October, 2007, Bill and Melinda Gates called together a prestigious group of malariologists, malaria R & D funding bodies and various international or, to use the newer buzz term, global health personages. Speaking before this group they articulated their goal, a global goal, to not just control malaria, but to eradicate malaria. This was an audacious challenge posed to the international community, but ever since the stars have been lining up to reassess and push research directions forward with this goal in mind at an expedited pace [14].

The especially good news, if this is to be achieved this time, is that malaria eradication means eliminating 'all' types of malaria, everywhere. Earlier eradication efforts were far from global, basically by-passing Africa, as well as various countries and regions around the world. Therefore, this manifesto was ground-shaking and meant that attention must start to realistically focus on *P. vivax *alongside continued work on *P. falciparum*, and eventually also include, the least prevalent human malaria species, *Plasmodium malariae *and *Plasmodium ovale *[15]. As noted above, the global widespread predominance of *P. vivax *infections outside of Africa and this parasite's special adaptive features, such as the presence and activation of dormant liver-stage forms called hypnozoites (more on this topic below) makes it a fierce and tenacious enemy, with debilitating health and socioeconomic ramifications for families, communities and nations. Thus, the proclamation to eradicate malaria is great news for the tens of millions, or, by the higher estimates, several hundred million, people who become infected each year and suffer dearly with *P. vivax *infections.

Over the past few decades, for all aspects of *vivax *malaria research, there have been a restricted number of people studying this parasite and disease, and thus very few people have been honing expertise on *P. vivax*. However, in the past few years, the number of researchers working to improve knowledge on the biology, epidemiology, clinical features, and treatment of *P. vivax *has been increasing, or perhaps just becoming increasingly visible and outspoken. Thanks to the Multilateral Initiative on Malaria, and other supporters, two international conferences were organized in 2002 and 2005 with an exclusive focus on *P. vivax*. Other recent meetings of MALVAC, a malaria vaccine advisory committee of WHO/IVR, focused on *P. vivax *vaccine development in 2005 (Cali, Colombia) and in 2007 (Barcelona, Spain). In addition, there has been some excitement and anticipation building over the last few years with the development and completion of the *P. vivax *genome sequencing project [16]. The recent meetings and political efforts revolving around the genome project have aimed to demonstrate the need for increased emphasis on this parasite species, with a contingent of researchers eager to assist, and, importantly, to chart the way for post-genomic discovery and research directions. Today, the outlook and capabilities for research on *P. vivax *and envisioning *vivax*-specific preventive and curative tools are promising, but much more needs to be done. The Bill and Melinda Gates Foundation (BMGF), its partners such as the PATH Malaria Vaccine Initiative (MVI), and other traditional funding bodies (e.g. government agencies) can be confident in seeing strong progress from their investments in this research, especially if necessary strategic support mechanisms are provided.

So, what's next? As has been realized with the new call for eradication of malaria, new methods and strategies of intervention are required to block transmission and reduce carriers of *vivax *malaria. This will come through the results of focused research on the shared, unique, and unknown biological, clinical and epidemiological features of *P. vivax*. These special features of *vivax *malaria and the research needed to bring about new interventions have been discussed recently [2,3,16,17]. One of these interventions is, of course, a vaccine, or perhaps more correctly put, vaccines against *P. vivax *[17]. This review aims to bring heightened attention on this topic, critically assessing what *P. vivax *vaccine candidates have been in the pipeline, and what is required to rebuild this pipeline and successfully advance new candidates for preclinical and clinical trials.

### *P. vivax *– status of clinical vaccine trials today

Where does *P. vivax *vaccine progress stand amongst the myriad of development activities being undertaken towards vaccines to protect against *P. falciparum*? Well, historically, since the mid-1980s there has been very little specific focus on *P. vivax *vaccine development, and this fact is reflected in the malaria vaccine 'rainbow tables', with only a few *vivax *vaccine candidates listed among 56 preclinical programmes and 20 clinical studies [18]. Very few laboratories (they can be counted on one hand, two at most) have worked to identify and pre-clinically characterize *P. vivax *vaccine candidates, and only a couple of *P. vivax *antigens are imminently poised to advance into clinical trials. One is an asexual blood-stage antigen, the merozoite invasion ligand protein known as the Duffy Binding Protein (DBP), whose binding domain (RII) has gone through preclinical testing in rodents and non-human primates [19-24]. The pre-erythrocytic/sporozoite antigen, the Circumsporozoite Protein (CSP), also has under gone preclinical studies in mice and primates, testing various platforms and formulations to advance into clinical trials [25-28]. One other *P. vivax *candidate vaccine, a transmission blocking sexual stage antigen, Pvs25, has been in phase I clinical trials [29,30].

### The *vivax*-specific vaccine development pipeline (or lack of)

Below, the major *vivax *vaccine candidates and other antigens with vaccine potential are described and analysed with considering the published data and the potential hurdles that are unique to *vivax *malaria. Then, research needs necessary to move *P. vivax *vaccine development forward and not in the shadow of *P. falciparum *vaccine development are proposed. It must be remembered that *P. falciparum *and *P. vivax *are genetically distant malaria parasites and vaccination with comparable antigens for one species predictably will likely not provide protection against the other species or even necessarily provide the same role in functional immunity.

In general, the thrust of efforts for *vivax *blood-stage vaccine discovery and development efforts, albeit limited in scope, have mostly followed in the footsteps of what was first being accomplished in the *P. falciparum *vaccine developmental pathway. Thus, after the Merozoite Surface Protein -1 (MSP-1) was discovered as a major merozoite surface antigen in *Plasmodium yoelii *and *P. falciparum *[31,32], the orthologous gene for the *P. vivax *antigen was characterized [33]. Research led to the identification of the C-terminal end of PfMSP-1 as the target of inhibitory antigens [32], and the equivalent PvMSP-1-p19 and p42 portions were subsequently produced and examined as *vivax *vaccine candidates ([34], Barnwell, Longacre & David, unpublished data).

Other asexual blood-stage antigens of *P. vivax *that have worked their way through to some point of pre-clinical testing include, MSP-3 family members [35,36] and the Apical Membrane Antigen-1 (AMA-1) [37,38]. While they have been 'hopeful' candidates, like MSP-1 at some point in time, and still are in the view of many, these *vivax *malaria target antigens are currently not being pursued with vigor towards scheduled clinical trials. This is in part due to the fact that these antigens are direct counterparts of the presently most popular blood-stage vaccine candidates for *P. falciparum*, which are all presently in a series of clinical trials in areas endemic for *P. falciparum *and awaiting definitive outcomes. If successful for *P. falciparum*, then it might be expected to see these *P. vivax *antigens also advance into clinical trials. However, that prospect, based on early results of the *P. falciparum *vaccines, is not without some uncertainties at this time. Beyond the above stalwarts of malaria vaccine development there are not too many other serious candidates in the *vivax *vaccine pipeline undergoing preclinical investigations; Thrombospondin Related Anonymous Protein (TRAP), Reticulocyte Binding Proteins (RBPs), and MSP-9 are among the few receiving some pre-clinical development effort [39-42].

### Asexual *P. vivax *blood-stage vaccine candidates

#### Duffy Binding Protein

The interaction of the Duffy Binding Protein (DBP) at the apical end of the merozoite with its red cell receptor, the Duffy blood group antigen, also known as DARC (Duffy Antigen Receptor for Chemokines), is essential for *P. vivax *to invade human red blood cells [43,44]. The *dbp *gene is a paralog of *eba-175 *and four other genes of *P. falciparum *encoding ligands that are important for invasion by that species, but in *P. vivax *there is a single gene within this Duffy-Binding like (*dbl*) family [45-47]. This lack of alternative *dbl *gene family members in the *P. vivax *genome and the almost universal dependence of *vivax *merozoites on DARC for entry into RBCs [48,49] has made the DBP a favourite, if not *the *favourite, candidate for a *vivax *blood-stage vaccine [43]. Since the identification of the *dbp *gene, a conserved cysteine-rich binding domain (Region II) has been mapped [20,50,51] and the function and structure has been further evaluated using *Plasmodium knowlesi*. Studies with transfected parasites lacking the *pkdbpα *gene, the ortholog of *pvdbp*, show that the DBP is important for junction formation between the merozoite and human red blood cells [52]. The PkDBP X-ray crystallographic 3-dimensional structure indicates the binding domain is conserved structurally [53]. Immunological assays in conjunction with binding assays are mapping the important polymorphic sites that may affect the functionality of antibodies raised by active immunization and natural infection [54-58]. Continued studies assessing PvDBP-RII polymorphisms in different geographical locations and in regards to functional immunity and the effects of polymorphism on protective efficacy will be warranted to continue to support its place as a leading *P. vivax *vaccine candidate.

The *Escherichia coli *expressed and re-folded Region II binding domain of the DBP [59,60] has gone through preclinical testing in rodents and non-human primates [21-24]. In an immunogenicity study, the *E. coli *expressed and re-folded PvDBP-RII candidate was used to immunize *Macaca mulatta*, rhesus monkeys, which showed strong and sustained antibody responses when formulated with Alum, AS02A or Montanide ISA 720 [23]. As the rhesus monkey is a primate host that does not accept infection with *P. vivax*, the objective in this case was adjuvant selection and not determining protective efficacy, although the induced antibodies did show high-titered blocking of PvDBP-RII binding with DARC by *in vitro *assay [23]. A prior study reported the immunization and challenge of small New World monkeys, *Aotus griseimembra *(owl monkeys), with PvDBP-RII formulated with the powerful Freund's Complete Adjuvant (FCA) [22]. These monkeys can be infected with *P. vivax *and use the Duffy antigen as a receptor for invasion [20], however, despite the hint of protection shown (delay in patent infection and reduction in cumulative parasitaemia), the results are not convincing with regards to overall protective efficiency. Because the *P. vivax *parasitaemia in both control and vaccine groups in the challenged monkeys were all at low levels and fairly erratic in this host, likely are due to the use of a line of the Salvador I strain of *P. vivax *that is not completely adapted to a spleen-intact host, it is difficult to accurately assess the biological effects of this vaccine in this model of *vivax *malaria [22]. Besides, caution is also warranted in the interpretation because FCA is known to be an exceptionally powerful adjuvant, but it is unacceptable for human use due to necrotic lesions at the injection sites and other complications; this adjuvant is also not currently acceptable for use in monkeys for the same reasons. While this vaccine candidate formulated with FCA nonetheless gave a hint of protection in this host, warranting further interest, it should be noted the *Aotus *monkeys receiving PvDBP-RII formulated with Montanide ISA 720, which has been used in humans, showed little or no protection [22].

The PvDBP-RII vaccine candidate is now being developed for clinical trials pending a reformulation with a suitable adjuvant and satisfactory re-testing in pre-clinical trials. The DBP RII antigen is known to induce the production of antibodies in a variety of species that by *in vitro *assay block adhesion of red blood cells to the DBP [22-24,57] and antibodies from immunized small animals and infected humans apparently tend to show inhibition of merozoite invasion *in vitro *in a field assay that produces low invasion rates [57,56]. Whether or not immunization of humans with this antigen will result in antibodies that will effectively inhibit the invasion of red blood cells by merozoites *in vivo *and, therefore impair parasite multiplication, remains to be shown. In the above mentioned trial the DBP antibodies were exceptionally high-titered in the partially protected *Aotus *monkeys, exhibiting strong inhibition of red cell binding at several magnitudes of dilution and yet the animals still became infected and parasitaemia levels increased after challenge. It must be remembered that the DBP is sequestered in the micronemes and not exposed to antibodies until probably just before or at the time of contact with the reticulocyte and at that time forms an irreversible bond with DARC and likely becomes part of the moving junction [52,61]. There is a short period of time for antibodies to act to neutralize the DBP/Duffy interaction and whether much of this invasion ligand or important epitopes are exposed in the moving junction to the host environment is not known. In this regards it has been suggested that the DBP be combined with another merozoite antigen, such as MSP-1 [21], but a better choice might be AMA-1 because this combination will concentrate the point of antibody attack at the machinery of invasion by neutralizing simultaneously two invasion ligands with different functions that are critical to parasite survival.

### Merozoite Surface Protein-1

*Plasmodium vivax *MSP-1, like *P. falciparum *MSP-1, exhibits allelic dimorphism as represented by the Belem and Salvador I strains, as well as other forms of diversity. [33,62-64]. As it also undergoes a similar pattern of primary and secondary proteolytic processing as *P. falciparum *MSP-1, approximately 42 kDa and 19 kDa C-terminal fragments are produced. *P. vivax *MSP-1p42 and MSP-1p19 vaccine candidates produced by baculovirus expression [35] have been tested in preclinical immunization trial conducted in *Saimiri boliviensis *(squirrel monkeys), a host susceptible to *P. vivax *infections, using FCA as the adjuvant (Barnwell, Longacre and David, unpublished data). The immunized squirrel monkeys produced tremendous titers of MSP-1 specific antibodies by ELISA on recombinant antigen (>5 × 10^6^) and by IFA on native free merozoites or matured schizonts (>1:40,000) of *P. vivax *(Belem strain). Sera from MSP-1p42 and MSP-1p19 immunized rabbits and monkeys inhibited *in vitro *merozoite invasion by 75–80% and *in vivo *peak parasitaemia was reduced by 80 to 90%, but there was no increase in the length of pre-patent periods from that of control animals after giving challenge infections of 100,000 Belem strain blood-stage parasites.

Another immunization trial in *Saimiri boliviensis *utilized yeast expressed MSP-1p19 formulated with alum and the nonionic block copolymer P1005 ([65,66]. The block copolymer P1005 was a potent adjuvant, inducing the highest antibody titers as measured by ELISA and IFA and a partial immunity where three of five monkeys had peak parasitaemia <50 parasites/μl but two others were > 20,000 μl. The other immunization groups (MSP-1p19 + alum and MSP-1p19 alone) and the control group were similar to each other with at least one monkey in each group of five or four, respectively, giving peak parasitaemia of < 1000 parasites/μl. However, here again, as noted above, a line of Salvador I strain previously passed in splenectomized animals was used in spleen intact monkeys, which were splenectomized one week after challenge with 100,000 parasites to allow parasite levels to increase to be microscopically patent by thick blood film (10 parasites/μl). Future trials should be conducted with *P. vivax *strains that are adapted to grow uniformily in non-splenectomized monkeys to be able to more reliably interpret results as the spleen is of central importance factor in malaria erythrocytic stage biology and immunity [67-72].

*Plasmodium cynomolgi*, a simian malaria parasite, is nearly identical in morphology and biology and genetically very closely related to *P. vivax *and in its macaque monkey hosts offer valuable models for the study of *vivax *malaria [73,74]. The structure of a *P. cynomolgi *counterpart of *P. vivax *MSP-1p19 has been determined by X-ray crystallography at 1.8 angstrom resolution [75]. This *P. cynomolgi *baculovirus expressed MSP-1p19 counterpart vaccine has been used for immunization trials in toque monkeys, *Macaca sinica*, a natural host of *P. cynomolgi *in Sri Lanka [76]. Significant levels of protection ranging from transient parasitaemia to completely negative outcomes were achieved in this model with FCA adjuvant formulations. Antibody titers by ELISA (>10^6^) and IFA (>5 × 10^4^) were sustained at high levels over the course of the study.

More recently, a PvMSP-1p42 *E. coli*-derived antigen was produced in two forms, soluble and insoluble, which was re-folded after solubilization, and used with Montanide ISA 720 as adjuvant to immunize rhesus monkeys [77]. Counterpart soluble and refolded *P. cynomolgi *MSP-1p42 antigens were also prepared and used to immunize rhesus monkeys using Montanide ISA 720 as adjuvant. Certainly, there were some surprises when the MSP-1p42 immunized monkeys were then cross-challenged with *P. cynomolgi *[78]. Statistically significant decreases of several parasitological parameters observed for each of the immunized groups as compared to the control group suggested a cross-protective immune response with this different, but genetically related parasite [78]. Surprisingly, however, though there is only a 75–80% identity between the *P. cynomolgi *and *P. vivax *antigens, there were no significant differences between any of the antigens used for immunization in the degree of protection provided except for the soluble *P. vivax *MSP-1p42 vaccine, which showed the greatest decreases in parasite burdens. Nevertheless, in this study all monkeys became positive, with no differences in prepatent periods and in the early acute phases of infection the rapid rate of parasitemic increases were the same as in the control group. Peak parasitaemia were reached somewhat sooner in the immunized animals than in control monkeys with a reduction of 60 to 75%. This picture of no discernable effect on early parasitaemias with a protective immune response kicking in after a period of active infection is a phenomenon seen with most MSP-1 immunizations, whether with *P. falciparum*, *P. vivax *or *P. cynomolgi*. At this time there is no indication that this vaccine formulation is being positioned for inclusion in a clinical trial.

A third *P. vivax *MSP-1 vaccine candidate antigen representing a 359 amino acid N-terminal portion of the Belem strain MSP-1 is known as Pv200L. It was produced in *E. coli *and the purified antigen was tested for immunogenicity and efficacy in *A. griseimembra *[79]. Not unexpectedly, the Pv200L recombinant antigen when formulated in FCA was highly immunogenic producing very high ELISA titers (>10^7^), but only modest (1:2,000) IFA titers. Despite the extraordinarily high specific antibody levels as measured by ELISA, there were no statistically significant differences between the vaccine immunized and control animals, again with peak parasitaemia and other parasitic parameters being relatively low in this model system using the Salvador I strain of *P. vivax *as the challenge parasite in owl (*Aotus*) monkeys [79]. Nevertheless, one prospective study has indicated there is a reduced risk of infection and clinical protection associated with antibodies to the N-terminal region of PvMSP1 [80], which is contrary to the prevailing conventional wisdom that the C-terminus of MSP-1 is the principal target of protective antibodies.

### Apical Membrane Antigen-1

Soon after its gene was characterized in 1997, the immunogenicity of an AMA-1 yeast product in rhesus monkeys was reported in 1999 [38]. The GSK adjuvant, AS02 was used with the antigen for immunization and ELISA titers were >2 × 10^5 ^and IFA titers were >1:10,000 after the third immunization. These animals were also challenged with *P. cynomolgi *and unlike in the case of MSP-1p42 noted above this cross-species challenge did not show any differences in parasitemic factors in the AMA-1 immunized group compared to the control animals. Basic studies have continued to show 1) its structure by X-ray crystallography [81,82], 2) phylogenetic structural comparisons to AMA-1 orthologs in other species [83], and 3) a few surveys of polymorphism or immunogenicity in Asia and the Americas [84-86]. However, there seems to be little further efforts in pre-clinical development to elevate it to phase I clinical testing at this time. As with MSP-1, AMA-1 is a prime candidate for *P. falciparum *blood-stage candidates and several *P. falciparum *products are in phase I and phase II clinical trials in the field. An efficacy trial in a suitable monkey model for *P. vivax *(see below) could likely provide the kind of data that is needed to drive this candidate into phase I and phase II clinical trials, if the ongoing clinical trials of *P. falciparum *AMA-1 prove to be promising.

### Merozoite Surface Protein-3

Another leading asexual blood-stage candidate vaccine antigen for *P. falciparum *is MSP-3, which is also undergoing clinical trials in Africa [87,88]. MSP-3 homologs also exist in *P. vivax*, and while similar enough to strongly indicate relatedness to PfMSP-3, the antigens expressed in *P. vivax *comprise a gene family with unique structural and antigenic characteristics [35,36]. In the Salvador I strain, the genome project has revealed eleven *msp-3 *genes of similar character in *P. vivax *distributed head-to-toe in a single locus [16]. All eleven genes encode putative proteins that share the same structural characteristics of a large alanine-rich central domain of heptad repeats forming coiled coils tertiary structure [35,36]. This gene family also shows considerable intraspecies variation characterized by numerous nonsynonymous SNPs and indels particularly in the central core domain [89,90]. Twelve years ago, immunization of *Saimiri boliviensis *monkeys with two products of the PvMSP-3 family (PvMSP-3α and PvMSP-3β) expressed in *E. coli *and using FCA induced strong immune responses in the form of antibodies as determined by IFA (titers of 1:10,240 to 40,960) and ELISA (titers >10^6^) (Barnwell and Galinski, unpublished data). Additionally, MSP-3α/β was shown to produce a modest level of protection by reducing peak parasitaemia by 60–70% and attenuating the course of infection in the spleen-intact squirrel monkeys infected with the Belem strain. Recently, immunization of *Saimiri boliviensis *with *E. coli *expressed recombinant 6His-PvMSP-3α formulated in Montanide ISA-720 did not confer protection (Jiang, Barnwell & Galinski, unpublished data). Considering the number of *msp-3 *genes potentially expressed in *P. vivax *merozoites and the allelic diversity demonstrated by this antigen family it may be an insurmountable challenge to develop a vaccine based on MSP-3 for *P. vivax*. On the other hand, some data suggest that one of the eleven *msp3 *genes may be over expressed relative to the others (Jiang, Barnwell & Galinski, unpublished data) and this could be a predominantly expressed family member.

### Other potential blood-stage vaccine candidates

Members of the Reticulocye Binding Protein-Like (RBL) family of proteins certainly should be considered as potential candidates to be included in malaria blood-stage vaccines as an important set of invasion ligands functionally expressed by merozoites [44,91]. *Plasmodium vivax *preferentially invades reticulocytes presumably by the selective attachment of members of the Reticulocyte Binding Protein (RBP) family, such as PvRBP-1 and PvRBP-2 [6,40,41]. The use of short-term cultured *P. vivax *schizont-infected RBCs for robust *in vitro *merozoite invasion inhibition assays [50], have shown that polyclonal antibodies raised against certain regions of PvRBP-1 and PvRBP-2 expressed in *E. coli *are capable of modestly inhibiting invasion of the *P. vivax *merozoite by 20–40% (Barnwell, unpublished data). The reticulocyte binding regions of PvRBP-1 and PvRBP-2 have been mapped to a region of the N-terminus of these large proteins (>300 kDa) through expression on the surface of COS cells (G. Rosas et al., unpublished data) and further pre-clinical investigation, such as optimizing in *E. coli *and/or yeast RBP subunit expression and further testing of structure-function requirements through reticulocyte binding assays as well as conducting non-human primate trials for immunogenicity, formulation and efficacy will likely guide whether this class of vaccine antigens will advance to clinical trials. Studies with patient samples are also beginning to provide relevant data regarding polymorphism and naturally acquired immunity to these proteins [92,93].

Additionally, the sequencing of the *P. vivax *genome has brought to light new *rbl *family members as potential players for pre-clinical evaluation as vaccine candidates. Their discovery may also help to understand the molecular mechanisms underpinning basic fitness requirements and alternative pathways of this parasite, such as the preferential invasion of reticulocytes through the attachment of members of the RBP family [6,40,41]. In addition to the two original RBPs (PvRBP-1 and PvRBP-2) there are at least five additional *rbl *genes in the genome. But caution is advised, since these new gene sequences represent both pseudogenes and *bona fide *expressed genes (Meyer, Barnwell and Galinski, unpublished data); this is a case in point, that although the *P. vivax *genome is available, annotation and validation of the 5,000+ genes identified need to now take center stage. One next step to progress the RBLs in the *vivax *vaccine pipeline is to complement the *P. vivax *genome database with regards to knowledge on the expression and interplay of the original and newly identified *rbl *members in *P. vivax*.

Another antigen for possible consideration as a *vivax *vaccine candidate is the Merozoite Surface Protein-9 (MSP-9) [42]. *Plasmodium vivax *MSP-9 is encoded by a highly conserved gene, and ortholog genes are present in *P. falciparum*, in rodent malaria species and in the simian malaria species, *Plasmodium coatneyi, P. cynomolgi*, and *P. knowlesi *[94]. The N-terminal region of MSP-9 is conserved and polyclonal or monoclonal antibodies will inhibit merozoite invasion [42,95]. Naturally exposed individuals have shown significant humoral and cellular responses, and in fact, an immunodominant epitope has been identified and further work will determine if this immunodominant epitope is capable of eliciting a protective response against *vivax *malaria [96].

It is made evident by the above summary of antigens and *vivax *blood-stage vaccine candidates that there is a severe need to keep the pump for the vaccine candidate pipeline well-primed. The sequencing of the genome of *P. vivax *has been a step in that direction and, in fact, this *vivax *genome database is already providing new genes and data that will very likely increase the pool of blood-stage vaccine candidates in the near future.

### Transmission blocking vaccines

The target antigens of transmission blocking vaccines (TBV) are exposed in the sexual stages of the parasite, the gamete, zygote and ookinete, in the gut of the mosquito after she has taken a blood meal containing circulating male and female gametocytes. Early studies on transmission-blocking immunity (TBI) indicated the primary mechanism of action was through antibody assisted by complement and more recent studies indicate that antibody-mediated blocking of oocyst formation is a reliable *in vitro *correlate of TBI [97-99]. The primary candidate antigens for TBV in *P. vivax *can be said to essentially be the same suspects as for *P. falciparum*: Pvs230, Pvs48/45, Pvs28 and Pvs25. Pv230 and Pv48/45 are still in waiting to be brought into preclinical studies primarily because of problems in efficient and structurally sound recombinant expression, although monoclonal antibodies have indicated in the past that these are important targets of TBI [100-102]. Despite the challenges facing the production of TBVs, their promise in adding a critical tool to the few available for control of malaria transmission is exceptional. But, at the current pace, this is like "*Waiting for Godot*".

### Pvs25

At this time, for *P. vivax *TBV, Pvs25 is by far the most advanced candidate having undergone extensive pre-clinical testing and development and it has been in early phase I clinical trials. Pvs25 can be produced efficiently as a recombinant in yeast [103] and earlier pre-clinical testing in mice and non-human primates had indicated that antibody responses could be generated that would cause a 70–100% reduction in both the number of infected mosquitoes and in oocyst burden in infected mosquitoes using adjuvants such as Montanide ISA 720 and Alum [99,104-108]. While Pvs25 antibody titers could generally remain elevated in New World primates, in rhesus monkeys maximum transmission-blocking titers required a second appropriately timed booster and then declined over the next few weeks and months [99,107,108]. In two clinical trials, one with an Alum formulation showed moderate transmission blocking antibody titers could be obtained with this adjuvant, but the second trial was curtailed due to reactogenicity of the antigen and Montanide ISA 51 adjuvant, which was deemed too frequent and severe including some with systemic reactions [29,30]. Further studies on increasing and sustaining Pvs25 transmission blocking antibody titers and providing safer, but potent adjuvant formulations are of immediate importance [109,110]. Another track for TBV development has been to use DNA vaccines encoding Pvs25 and Pvs28, which at least in mice has produced respectable blocking antibody titers [111]. It will also be important, in parallel, to speed up the pre-clinical selection of other new sexual stage antigens such as CTRP [112] and WARP [113] along with research on the expression of Pvs230 and Pvs48/45 to broaden and enhance the transmission blocking effectiveness of these vaccines that will be critical for increasing the chances of controlling and eliminating *P. vivax *malaria.

### Pre-erythrocytic (sporozoite and liver stage) candidate antigens and vaccines

Like the asexual blood stage, vaccine development targeting *vivax *malaria pre-erythrocytic stages has mostly concentrated on those orthologs of antigens already being studied for *P. falciparum *vaccine development. *Plasmodium vivax *pre-erythrocytic vaccines target two sporozoite antigens, the circumsporozoite protein (CSP) and thrombospondin-related anonymous protein (TRAP) antigens, which are conserved across many species of *Plasmodium*. Following in the foot-steps of *P. falciparum *CSP vaccine efforts, a nearly full-length PvCSP was first recombinantly synthesized in yeast in 1987 [114]. This PvCSP recombinant antigen and PvCSP repeat unit peptide vaccines were tested in a Bolivian squirrel (*S. boliviensis*) monkey-*P. vivax *challenge model providing little or no protective effects [115-119]. Clinical trials with yeast-derived PvCSP were disappointing and showed low immunogenicity and essentially mirrored results from the efficacy tests in New World monkeys [120]. Similar results were obtained with early *P. falciparum *CSP vaccines in clinical challenge studies in malaria naïve human subjects, until RTS, S, the chimeric PfCSP/Hepatitis B surface protein construct, gave partial protection lasting a few weeks to several months when combined with a saponin-based adjuvant [121-124]. *Plasmodium vivax *multiple antigen constructs (MACs) of CSP vaccines have shown some protection in New World monkeys against *P. vivax *sporozoite challenge showing 40% efficacy or less that waned over several months [125], which is similar to the efficacy reported in field trials for RTS, S. The multiple antigen peptide vehicles though have lost popularity because of the difficulties in providing batch-to-batch consistency. Nevertheless, in this non-human primate trial antibodies were produced against the AGDR B-cell epitope found in the repeat amino acid units of the VK-210 allele of the *P. vivax *CSP. This epitope was a correlate of protection in that a mouse monoclonal antibody recognizing this epitope was highly protective upon passive transfer in squirrel monkeys [118]. However, humans or monkeys previously immunized with *P. vivax *CSP vaccine constructs did not produce antibodies to the AGDR epitope although they recognized the repeat unit peptides [118,119].

Only recently have *vivax *malaria vaccine studies based on PvCSP been revisited since the initial studies first undertaken a decade or two ago. Unlike *P. falciparum*, *P. vivax *has two different alleles of the *csp *gene (VK210 and VK247 or type I and type II, respectively) with the encoded repeat amino acid unit differing in sequence between the two alleles [126]. Currently, PvCSP vaccine candidates are being made in the form of chimeric recombinant proteins expressed in *E. coli *and encoding both types of repeat units [29] or as three long synthetic peptides covering portions of the N-terminal, C-terminal and repeat regions of PvCSP (Salvador I strain) and directed to one allele [26,27]. Studies with the *E. coli *expressed chimeric recombinant protein are just beginning in non-human primate trials and early phase I trials are being planned. The long synthetic peptide vaccine formulated in Montanide ISA 720 has been through trials in both non-human primates (*Aotus l. griseimembra*) and a phase Ia clinical trial showing immunogenicity by eliciting good antibody responses (ELISA and IFA) and γIFN production, the latter particularly in response to live sporozoite exposure in owl (*Aotus*) monkeys [27]. However, in each case, human and non-human primates, challenge with live *P. vivax *sporozoites was not reported.

A long synthetic peptide of a region of PvTRAP has also been synthesized and in pre-clinical studies was used to immunize *A. griseimembra *monkeys, which were subsequently challenged with a wildtype isolate of *P. vivax *sporozoites acquired by feeding *Anopheles albimanus *mosquitoes on a patient [127]. Although four out of six control monkeys became positive for blood-stage parasites and only two out of six developed an infection in the vaccine group, the result was statistically not significant and must be interpreted that in this small trial no protection was demonstrated. This result, though, is also similar to results for PvCSP vaccine constructs in past clinical trials and non-human primate immunization studies noted above.

Although called pre-erythrocytic vaccines, most development has concentrated on the abundantly expressed surface protein of sporozoites, CSP, which is important in motility and hepatocyte invasion [128]. First described as an immunodominant antigen, it is becoming clear from past vaccine studies and more recent investigations that the CSP alone will induce a level of immunity, but this immunity is incomplete and leaky [124,129]. The CSP thus is a functionally competent component of a pre-erythrocytic vaccine, but it is likely going to be insufficient to generate a solid immunity as would be required for this type of vaccine and other liver-stage antigens are likely needed to generate the solid immunity exhibited by attenuated sporozoites [130]. Recently, there has been a popular notion that combining a pre-erythrocytic component with an erythrocytic component will produce a more efficacious malaria vaccine. Given the mutually exclusive immunity generally obtained between the pre-erythrocytic and erythrocytic parasite stages, it is unlikely that combining two antigens which each generate partial, incomplete immunities against their respective developmental forms will necessarily improve upon the overall protective efficacy of a vaccine. Whether the parasite load in the liver is effectively reduced 30% or 90% in an immunized individual, one or two escaping liver schizonts will produce thousands of merozoites igniting an acute blood-stage infection to be handled by another partially effective immune response. Perhaps, it would be better to combine CSP with other sporozoite or liver-stage antigens to make a pre-erythrocytic vaccine that is much more of an effective barrier on liver-stage development and then similarly combine selected merozoite surface and ligand antigens to achieve a more effective blood-stage vaccine.

### Live, attenuated sporozoite vaccines

A major effort is now being made to bring about a *P. falciparum *pre-erythrocytic vaccine based upon either radiation or genetically attenuated sporozoites [131,132]. Experiments performed over thirty years ago with radiation attenuated, live sporozoites, which, by the way, were injected by living, feeding mosquitoes, were the first direct proof of principle that a human malaria vaccine could be feasible [133]. However, in these past studies, less than a couple of dozen individuals were fed upon by irradiated, *P. falciparum *or *P. vivax *sporozoite infected mosquitoes and 16 had been observed to be solidly protected upon challenge with normal fully infective sporozoites [133-135]. In these 16 individuals, protection apparently lasted between 2.5 and 10.5 months for *P. falciparum *and around six months for *P. vivax*. Indeed, for *P. vivax*, the number of immunized and protected individuals sensitized through the mosquito bite injection of sporozoites was a grand total of two.

A *P. vivax *attenuated sporozoite vaccine is certainly being considered, but poses a number of challenges. Most importantly, a major challenge would be the limited, or in reality, non-existent sources for reproducibly acquiring standardized *P. vivax *sporozoites for attenuation. This is worth noting given the lack of a practical continuous culture system that would provide infective gametocytes or the ethical considerations that surround the ability to infect humans with cloned lines of *P. vivax *or a genetically altered line of parasites in order to feed mosquitoes and produce infected mosquitoes with attenuated sporozoites. Efforts are being made to develop a human challenge system for *P. vivax *to evaluate *vivax *malaria vaccines by feeding upon patients with *vivax *malaria in Colombia and Thailand, but this has its drawbacks, even as a challenge system, as the parasites will be a heterogeneous mixtures of genotypes and it will be difficult to sort out results of vaccine trials. This is particularly true as immunizations will be based upon antigen alleles likely to differ from those present in the challenge inoculum.

While prison volunteers were infected with *P. vivax *and fed upon to infect mosquitoes in the past [73,136], this practice has since been judged unethical and it is not likely that at present anyone will convince ethically suitable volunteers to go through "benign" *vivax *malaria infections long enough to efficiently infect large numbers of mosquitoes that will produce heavy loads of sporozoites. Although chimpanzees were used to infect tens of thousands of mosquitoes with billions of sporozoites from various strains of *P. vivax *in the past, they are no longer generally available for these purposes due to restrictions imposed by current ethical considerations for the treatment and use of great apes.

Clearly, a cataloguing of what genes in the *P. vivax *genome are expressed during hepatic stage development needs to be generated in order to adequately develop genetically attenuated sporozoites or, more urgently, to advance recombinant sub-unit pre-erythrocytic vaccines. Two products, LSA-1 and LSA-3, identified from genes expressed during parasite development in the hepatocyte in *P. falciparum *are candidates for *falciparum *pre-erythrocytic malaria vaccines [137,138], but these genes apparently do not exist in *P. vivax*. Other liver-stage genes specific for *P. vivax *need to be discovered and exploration in this area will be facilitated given the availability of the *P. vivax *genome [16]. Identifying liver-stage antigens that might be most useful for generating effective recombinant protein based or viral-vectored vaccines, which may or may not include CSP or TRAP components should be the priority. Albeit, in the near term, there will be difficulties in generating necessary and reliable post-genomic data on hepatic-stage parasites to identify these parasite products if the appropriate non-human primate animal model systems and associated expertise are not developed, maintained and utilized. This includes, most importantly, the study of the enigmatic hypnozoite.

### Hypnozoites and liver stage biology: a challenge to malaria vaccines

The most outstanding feature of *P. vivax *biology in regards to a challenge for control interventions is the ability of this parasite to form dormant hypnozoites in the liver, which when reactivated weeks, months or years later create new blood-stage infections through a true liver-stage relapse mechanism [139,140]. In regards to immunity, whether acquired naturally or actively through induction by immunization, this metabolically quiescent stage poses a challenge to vaccines directed against *P. vivax*. Next to nothing is known about the bio-physiology of this stage of the parasite and certainly minimally about the immunological recognition of and immune response to hypnozoites in the liver.

Hypnozoites, as the mechanism behind relapses, are found in *P. vivax*, presumably *P. ovale*, and the monkey malarias, *Plasmodium simiovale*, *Plasmodium fieldi *and *P. cynomolgi*, all of which are very closely related to *P. vivax *[73,74,139,141,142]. First coined in 1977 [143], it was not until 1980 that hypnozoites were identified in the liver tissue of a rhesus monkey injected with millions of sporozoites of *P. cynomolgi *[144,145] and, shortly thereafter, in a chimpanzee inoculated with *P. vivax *sporozoites [146,147]. These forms appear as nondescript small intra-hepatic round single-nucleated bodies about 4.5 μm in diameter. But what is a hypnozoite in terms of its metabolic activity and immune recognition? This is not known but obviously to remain in a hepatocyte, perhaps for a year and more, it must simultaneously exist under stasis with reduced metabolic activity and yet control and alter its host cell (inhibiting apoptosis?). Only primaquine and related 8-aminoquinolines are known to attack and kill hypnozoites although there are other antimalarial drugs that will destroy primary exo-erythrocytic schizonts providing radical cure in non-relapsing malarias [148,149]. This quiescent liver stage could similarly pose a problem for any pre-erythrocytic stage vaccine; by presenting a low metabolic and antigenic profile allowing the parasite to stealthily slip past the defenses of an immunized host, as they wane or fail to react in time when the parasite is reactivated. It is also known that relapse parasite populations produced from reactivated hypnozoites are quite often antigenically distinct from the primary parasitaemia and previously relapsed infections [150,151], possibly allowing a breakthrough relapse population to escape restricted immune responses induced by a blood-stage vaccine. Many of the obvious questions regarding *P. vivax*, hypnozoites, vaccines and immune recognition could be explored now since robust model host-parasite systems such as *P. cynomolgi *or *P. simiovale *in rhesus macaques exist [142,152-154] or, for that matter, a few specific *P. vivax *strains that permit relapse infections can be observed and studied in *Aotus nancymaae*.

Out of six squirrel (*Saimiri boliviensis*) monkeys, each receiving intravenously 1.5 million *P. vivax *sporozoites irradiated with 15 krads, only two were fully protected upon challenge with live, virulent *P. vivax *sporozoites [115]. Since, on the other hand, only two humans have been immunized with radiation-attenuated sporozoites of *P. vivax*, it is difficult to compare results between non-human primates and human clinical experiments. Nevertheless, only two monkeys were protected and in this model system it is not known if hypnozoites were also eliminated along with primary schizonts, as relapse parasitaemia, unfortunately, has never been observed in this model of *P. vivax *and, therefore, it is unknown if viable hypnozoites ever existed.

*Plasmodium cynomolgi *in rhesus monkeys, an excellent surrogate model for *P. vivax *in humans, has not been adequately utilized to sort out and address the many questions posed by hypnozoites and their impact on acquired immunity. If effective protection can be induced by an attenuated or sub-unit pre-erythrocytic vaccine against primary liver trophozoites and schizonts of *P. vivax *(or *P. cynomolgi*) will that vaccine provide protection against hypnozoites that may reactivate weeks, months and sometimes year or more later? The one and only immunization trial undertaken to immunize macaque monkeys against *P. cynomolgi *pre-erythrocytic stages used 70,000 and 125,000 irradiated (13 krads) sporozoites injected intravenously into two animals, which upon challenge were only partially protected as shown by delays in patent infection of two and five days compared to one control monkey [155]. Given this experiment was carried out with only three monkeys, it is evident more studies need to be done. Using reliable primate malaria models it will be critical to have indications of whether pre-erythrocytic vaccines when effective against the primary tissue stages will also be effective against the relapse causing hypnozoites to prevent further relapses or dramatically reduce the number of relapses. Sub-optimal doses of radiation (6.5 to 8.5 krads) will reduce the number but not the pattern of relapse episodes in *P. cynomolgi*, just as will reducing the number of sporozoites inoculated in *P. vivax *or *P. cynomolgi*, [156,157]. However, trying to determine this kind of data, ie, whether a vaccine is preventing or reducing relapse rates, from human trials, especially in field settings, will present many obstacles and challenges that might not be readily overcome, if ever.

### Rebuilding a pipeline of candidates undergoing pre-clinical evaluations to reignite the advancement of *vivax *vaccines into clinical testing

In the current time-frame of malaria vaccine research, HIV vaccine research has taken a number of steps back along the pathway of development and many now advocate a need for further basic research prior to more clinical vaccine testing [158]. Malaria vaccine researchers may also be drawing close to the same line of action, if present *P. falciparum *vaccine candidates in clinical trials continue to not fulfill their expected promise. Although this will be less shocking as malaria researchers have knowingly remained too optimistically focused on a few antigens, while being keenfully aware of the much larger genome and complex biology of *Plasmodium*. It would certainly seem to be prudent sooner than later to vigorously again prime the pipeline with new vaccine candidates to consider and evaluate pre-clinically. This is especially true for *vivax *malaria vaccine development where, as we have noted above, there are really only two or three *P. vivax *vaccine candidates heading into early clinical trials and very few others in the pipeline undergoing serious preclinical evaluation with any adequate form of support. Simply put, more candidates need to enter this pipeline and the genome is ripe with potential targets.

Certainly, the sequencing and initial annotation of the genome of *P. vivax *[16] is one development that will greatly facilitate the feeding of the *vivax *malaria vaccine pipeline. There is yet much to be learned about the specific biology and the intricate details of the genes that determine the life cycle and host interactions of *P. vivax *and the functioning of its large genome comprising over 5,000 genes. Consequently, it is with high expectations that out of this genomic database relevant vaccine (and drug) interventions will come to light, and thus new candidates for *vivax *malaria vaccines will be revealed. With that said, the *vivax *vaccine development field now faces the challenge of post-genomic deciphering of the data now available from the *P. vivax *genome (Sal I strain) sequence. However, to accompany this (and future) genomic information reliable and relevant transcriptome, proteome and other types of 'ome data for the various life stages of *P. vivax *will be important to realistically mine this information [159], the provision of which may have more challenges than realized.

Of course, it has become clear to some that simply targeting the predominant surface coat proteins of sporozoites and merozoites, which were identified long ago in the wake of molecular technological approaches for vaccine development, may not reliably result in effective malaria vaccines capable of inducing high levels of protection. There will indeed be benefits from defining and understanding immunological correlates of protection, and dedicating more effort to testing new robust adjuvants to help achieve protective immune responses. Although this applies to malaria vaccines in general, in *vivax *malaria it is of considerable and central importance because of the lack of a practical *in vitro *system of continuous cultivation and a more dependant reliance on the use of human clinical cases and non-human primates for propagating the various developmental forms of *P. vivax *along with the relevant simian malarias and investigating host and parasite interactions.

### *P. vivax *and the *in vitro *'crockpot'

As much as it has been desired, and various rigorous efforts have been made towards this goal over a long period of time starting in 1912 [160], there is still no reliable, practical system for the long-term culture of the asexual blood and gametocyte stages of *P. vivax*. *In-vitro *culture of *P. falciparum *first became a reality in 1976. While there has been a productive method in use for the short-term culture of *P. vivax *with reinvasion of human or non-human primate reticulocytes since 1989 [49], *P. vivax *after the initial round of positive reinvasion tends to rapidly decrease in numbers and invasion efficiency with subsequent rounds of invasion even when fresh reticulocytes are supplied [161,162]. The most successful attempt with *P. vivax*, along the lines of current culture techniques for *P. falciparum*, was for 12 days of positive growth with an average two-fold multiplication rate, requiring media changes twice a day and a fresh supply of blood highly enriched in reticulocytes every 48 hours; clearly a difficult task [163]. This effort has not been successfully repeated since, although recently cord blood has been used to supply reticulocytes with a very low level of parasites maintained for one month [164]. More recent culture attempts have utilized culture systems of haematopoietic tissue and stems cells to continuously produce a lineage of erythroid developmental forms that include reticulocytes, which are then supplanted with *P. vivax*-infected erythrocytes from patients to initiate the continuous *P. vivax *cultures [165]. In general, although the *vivax *parasites in some cultures lasted over 30 days (>15 growth cycles), with one up to 85 days, the levels of parasites remained very low and culture maintenance is relatively expensive.

Improvements in these approaches are certainly needed and based on initial results further attempts are warranted. In the absence of a continuous culture for *P. vivax *blood stages, other more recent experiments to guide vaccine development have utilized infected blood from patients in short-term cultures of one growth cycle to perform merozoite invasion or growth inhibition assays [57]. But this method of evaluating vaccine targets, because of a lack of robust merozoite reinvasion and the genetic differences in the parasite populations that are used with each culture assay, is problematic and made less desirable. *In vitro *systems for production of infective gametocytes, certainly critical for *P. falciparum *TBV and PEV development, will also be critical for *vivax *malaria vaccine development, but will, naturally, be dependent on success with asexual blood-stage culture development. Of course, there still could be other challenges even with a robust asexual culture methodology for *P. vivax *given observations that gametocyte production is lost on continuous culture of *P. knowlesi *and *P. cynomolgi *[166,167], parasite species kindred to *P. vivax*.

### *Plasmodium vivax*, non-human primates and *in vivo *test tubes

In the absence of *in vitro *culture, the only available sources of material to directly investigate the genetics, biology, metabolism, immunity or pathology of *P. vivax *are infected humans and New World monkeys. Until recently, chimpanzees have been used to study *P. vivax *liver stages and provide thousands of mosquitoes heavily infected with sporozoites of *P. vivax*, but recently with increasing concern for using chimpanzees in research these kinds of valuable studies have declined and ceased. Many clinical studies, primarily limited to endemic regions, are able to utilize parasites collected from patients to study genetics or, as recently published, to develop a transcriptome profile [159] or for TBI and mosquito infectivity [106,168,169]. Certainly, many aspects of immunity and pathology are also desirably investigated in the intermediate host of *P. vivax*, humans. However, complete reliance on this source can present challenges and problematic circumstances. Fortunately, *P. vivax *can infect and be adapted to a number of species of New World monkeys.

Over the past two or three decades, a significant number of isolates of *P. vivax *(>40) have been partially or fully adapted to infect and grow well in various species of New World monkeys such as *A. l. griseimembra*, *A. nancymaae *and *Saimiri boliviensis *to name the most propitious hosts. These model systems of *P. vivax *infection will vary by the particular host and parasite strain combination with regards to what biology they are best suited to study. They have been used to infect mosquitoes for sporozoite production, *in vivo *challenge infections with sporozoites, and provide viable parasites for genetic transformation [170] and various immuno-biological and pre-clinical vaccine studies.

New World primate models that have been developed to evaluate pre-erythrocytic vaccines include selected strains in the compatible hosts, such as Brazil VII or Panama I in *Aotus nancymaae *or Salvador I in *Saimiri boliviensis *[117,171,172]. Other strains of monkey adapted *P. vivax *such as Belem and Palo Alto (Vietnam IV) because of consistent and high parasitaemia in non-splenectomized hosts are particularly suited for testing blood-stage vaccines [173]. *P. simium*, a natural adaptation of *P. vivax *in South American howler and spider monkeys can be used in either *Saimiri *or *Aotus *species to evaluate either pre-erythrocytic or blood-stage vaccines [174].

Moreover, the malaria parasites most closely related to *P. vivax*, *P. cynomolgi *and *P. simiovale*, are likely to be slated for genome sequencing in the near future along with new strains of *P. vivax*. Thus, the potential for increasing knowledge about *P. vivax *and investigator's capabilities for identifying new vaccine targets is at this time very high if model systems are also supported along with efforts to develop *in vitro *culture systems. Importantly, in the case of *P. vivax*, the simian malaria models, such as *P. cynomolgi *and *P. simiovale*, have historically served as crucial aids to decipher *P. vivax *biology and identify functionally important proteins. Without these primate host models for *P. vivax *and the very close kin relationship of the simian malaria, *P. cynomolgi*, in macaques, a lot less would have been accomplished over the past several decades in ongoing attempts to formulate a knowledge base on this neglected parasite.

Recently there has been a trend to use "humanized" mice to study blood-stage *P. falciparum *[175] and there are hopeful attempts to be able to include the pre-erythrocytic stages by transplanting human liver tissue in these highly immuno-compromized mice [176]. Similarly, there are designs to attempt to infect these "humanized" mice with the blood stages and sporozoites of *P. vivax*, but one has to really question whether these "models" represent solid experimental tools or are merely expensive *in vivo *test tubes of living tissues and organs that will be of narrowly targeted and limited value, which may also not provide credible data. An interesting twist, though, on using a rodent malaria model to study *P. vivax *is the recent genetic transformation of *Plasmodium berghei *that created a chimeric parasite by exchanging *P. berghei s25 *with the *s*25 gene from *P. vivax *to analyse TBV antibodies against this human parasite [177]. However, an alternative and, perhaps, a more promising avenue to study *P. vivax *vaccines and to decipher this species' biology would be to use genetic exchange transformation of *P. cynomolgi *or *P. simiovale *with *P. vivax *genes, or elements, as has been done for *P. knowlesi *with the *P. falciparum *CSP gene (C Kocken, personal communication).

### Research agenda for *P. vivax *vaccines

Because *vivax *research has been neglected for decades, the research needs agenda can look like a large wish list. However, the truth is that with today's technologies and eager scientists who are willing to collaborate and coordinate, much headway can be made rather quickly, assuming the provision of resources. Decades ago, prior to the genome era, research was slow and laborious, perhaps akin to the use of typewriters before the computer age and the Internet. Today, researchers can move quickly in unchartered territory, and the use of shared biological and electronic resources and data will expedite discovery. Rather than develop vaccines with a first come first serve approach, in reference to the top 10 proteins revealed through traditional methodologies, experts can really aim to find the Achilles heel, the most essential and vulnerable target sites of this parasite species. In this vein, one needs to know thy parasite better by also focusing on its unique features, including the hypnozoite, the reticulocyte host cell preference, and the specialized caveolae vesicle complex (CVC) structures it makes as it takes over the red blood cell, for both asexual and sexual stage progeny. There is also the need to better understand the pathogenic features, transmission cycle differences, and vector biology peculiarities compared to *P. falciparum*, as there are bound to be lessons that are relevant for the consideration of *vivax *vaccines, as well as future multi-species vaccines, which ultimately may be the best way to eliminate and ultimately eradicate this disease.

• Additional genome sequencing, transcriptome, proteome, structural biology, and other types of integrated systems research should be a priority. Without question, additional *P. vivax *and complementary simian malaria genomic data will help to reveal the genes and proteins of critical importance to the biology of these parasites. Post-genomic information is especially needed for pre-erythrocytic and sexual stages.

• Studies on the biology of *P. vivax *and the simian malaria parasites should be emphasized, with cataloguing of characteristics that are in common with *P. falciparum*, but, perhaps more importantly, those that are unique to *P. vivax*. Of major concern to many, the *P. vivax *liver-stage forms will be exceptionally challenging to study and this area of research is in need of dedicated resources. In addition, *P. vivax *researchers lack a continuous *in vitro *culture system to propagate blood-stage parasites. However, in the face of these challenges, there can be greater coordination and expanded use of *P. vivax *(and *P. cynomolgi*) infections in non-human primates to help circumvent these drawbacks. It remains uncertain if the development of a reliable continuous *in vitro *culture will be feasible, or not. Meanwhile, non-human primate infections can provide both liver-stage and blood-stage parasites for in-depth characterization and the identification of new *vivax *malaria vaccine targets. Despite the limitations in studying blood samples from patients, small volumes of blood from field samples can also continue to provide important genetic and biological information and opportunities for working in the field need to be expanded.

• Strategic directions and collaborations are then needed to funnel target candidate antigens and approaches through an express vaccine pre-clinical pipeline. Contrary to research as usual, we must be able to 'let go' of favourite antigens, platforms, etc.

• Researchers must crack the code of the hypnozoite, a black box at the moment, which can also be viewed as a Pandora's Box. Hypnozoites will continue to hide away and produce illness, if not tackled head on. Malaria eradication may be unreachable if hypnozoites are not better understood and eliminated via vaccination or a new effective drug for radical cure.

• Models, models, models! These are so much needed for *P. vivax *vaccine research, both *in vitro *and *in vivo*. Investment is needed to establish and improve upon possible model systems, and, importantly, keep honing the specialized expertise needed to work with non-human primates, malaria infections, and pre-clinical vaccine trials.

• Training, training, training! What is not accomplished in the next decade, in the aim to eradicate malaria, must be handed down successfully to future generations to continue in these steps, or humanity must accept that once again some have dreamt too big and left another historic note on how the world '*tried*' once again to eradicate malaria.

## Competing interests

The authors declare that they have no competing interests.
